# Metarhizium Anisopliae Challenges Immunity and Demography of *Plutella xylostella*

**DOI:** 10.3390/insects11100694

**Published:** 2020-10-13

**Authors:** Junaid Zafar, Rana Fartab Shoukat, Yuxin Zhang, Shoaib Freed, Xiaoxia Xu, Fengliang Jin

**Affiliations:** 1 Laboratory of Bio-Pesticide Creation and Application of Guangdong Province, College of Plant Protection, South China Agricultural University, Guangzhou 510642, China; jz_jaam@yahoo.com (J.Z.); ranafartab@gmail.com (R.F.S.); listzhang@163.com (Y.Z.); xuxiaoxia111@scau.edu.cn (X.X.); 2 Laboratory of Insect Microbiology and Biotechnology, Department of Entomology, Faculty of Agricultural Sciences and Technology, Bahauddin Zakariya University, Multan 66000, Pakistan; sfareed@bzu.edu.pk

**Keywords:** entomopathogenic fungi, genes, mortality, pathogenicity, demography

## Abstract

**Simple Summary:**

The diamondback moth, *Plutella xylostella*, is a destructive pest of cruciferous crops worldwide. Integrated pest management (IPM) strategies, largely involve the use chemical pesticides which are harmful for the environment and human health. In this study, the virulence of three species of entomopathogenic fungi were tested. *Metarhizium anisopliae* proved to be the most effective by killing more than 90% of the population. Based on which the fungus was selected to study the host-pathogen immune interactions. More precisely, after infection, superoxide dismutase (SOD) and phenoloxidase (PO), two major enzymes involved in immune response, were studied at different time points. The fungus gradually weakened the enzyme activities as the time progressed, indicating that physiological attributes of host were adversely affected. The expression of immune-related genes (Defensin, Spaetzle, Cecropin, Lysozyme, and Hemolin) varied on different time points. Moreover, the fungus negatively impacted the development of the host by reducing the life span and egg laying ability. Thus, *M. anisopliae* can become a potent prospect for the control of this pest. This information will also reinforce the development of policies for biocontrol-based pest management.

**Abstract:**

Entomopathogenic fungi are naturally existing microbes, that can serve as a key regulator of insect pests in integrated pest management strategies. Besides having no hazardous effects on the environment, these entomopathogens are alternatives to synthetic insecticides that can control notorious insect-like *Plutella xylostella*, a destructive pest of cruciferous crops. Three different species of entomopathogenic fungi were evaluated before the selection (high larval mortality and least LC_50_) of *Metarhizum anisopliae*. The study was designed to investigate the mortality, development, and immune responses of *P. xylostella* when challenged with *M. anisopliae*, a naturally existing soil-borne entomopathogenic fungus. *M. anisopliae* resulted in high pest mortality by killing 93% of larvae. However, no statistically significant effect on hemocyte concentration was observed. The activity of enzymes (Phenoloxidase and Superoxide dismutase) and immune genes (Defensin, Spaetzle, Cecropin, Lysozyme, and Hemolin) did vary at different time points (24, 48, 72 and 96 h) after exposure to *M. anisopliae*. Disturbance in the biological cycles of *P. xylostella* was also detected, significantly shorter adult life span (8.11:6.87, M:F) and reduced fecundity (101 eggs/female) were observed along with disturbed larval and pupal duration. Results suggest that *M. anisopliae* can efficiently hinder the *P. xylostella* defense and developmental system, resulting in mortality and disturbed demography.

## 1. Introduction

Entomopathogenic fungi are naturally existing microbial control agents that effectively regulate the insect pest populations [1,2]. Several entomopathogenic fungi have been used to control insect pests from different orders such as Diptera [3,4], Hemiptera [5], Coleoptera [6], Homoptera [7], and Lepidoptera [8]. The excessive and injudicious use of synthetic insecticides has resulted in pest resurgence, environment degradation, resistance development while also causing harmful effects to human health [9,10,11]. Such detrimental effects of chemical insecticides stressed the need to develop alternative control strategies. Furthermore, the resistance to insecticide curbed the control strategies, whereas insect pests are unable to develop resistance against entomopathogenic fungi making them an effective weapon against resilient pests [12,13,14,15,16].

*Plutella xylostella*, one of the most destructive lepidopteran pests of cruciferous crops, has caused severe economic damages (quantitative and qualitative), with an annual cost estimated to be USD 4–5 billion [17,18,19,20]. Over the years the pest has developed resistance against many control agents including dichloro-diphenyl-trichloroethane (DTT) and *Bacillus thuringiensis* (Bt) [21,22,23] making it difficult to control while also emphasizing the need to develop alternative control strategies. Among all the current strategies, biological control represents an eco-friendly approach with no hazardous effects on human health. Entomopathogenic fungi are biological control agents which are cosmopolitan in nature [1,24,25,26,27,28]. *Metarhizium anisopliae*, a soil-borne entomopathogenic fungus, represents an ecologically safe alternative to chemical pesticides [29]. The entomopathogen has proven to be effective against lepidopteran pests such as *Helicoverpa armigera* [30] and *Spodoptera exigua* [31]. *M. anisopliae* produces secondary toxins such as Destruxin A and E to repress the host immune responses while also deploying evasion protein such as Mcl1 protein (*Metarhizium* collagen-like protein) to avoid detection [32,33]. In response to the invasion of microbes, an array of recognition molecules detects the pathogen, resulting in the initiation of Toll and immune deficiency (Imd) pathways, that regulate anti-fungal and antibacterial defenses respectively [34,35]. Spaetzle, a gene encoding toll activating protease, initiates the immune pathway. Defensins are antimicrobial peptides responding to pathogenic challenges or injury. Similarly, cecropins constitute a major part of the insect innate immune system. Antioxidant enzymes such as superoxide dismutase (SOD), a key factor in host defense system, function in melanization and phagocytosis [36,37]. Likewise, Phenoloxidase (PO) is a key enzyme in the melanization cascade that also participates in cuticle sclerotization and wound healing [38,39]. In this study, we investigated the efficacy of *M. anisopliae* against *P. xylostella*. The present study aims to explore the interaction of entomopathogenic fungi with its insect host and elaborate the immune and developmental changes after the infection.

## 2. Materials and Methods

### 2.1. Insect and Fungi Culture

The population of *P. xylostella* was taken from the Institute of Plant Protection, Guangdong Academy of Agricultural Sciences, China by the Engineering Research Centre of Biological Control Ministry of Education, South China Agricultural University, Guangzhou, Guangdong Province, P. R. China. The colony was maintained in a pathogen-free environment. Larvae were kept at 25 ± 1 °C with a light: dark cycle of 16:8 h and 60–70% relative humidity [34]. Three different entomopathogenic fungi were obtained from the Laboratory of Insect Microbiology and Biotechnology, Bahauddin Zakariya University, Multan, Pakistan (Table 1), and screened against *P. xylostella*. To prevent aging, isolates were passage through the host [40]. Monoconidial culture (14 days) grown on potato dextrose agar (PDA) was harvested with a disinfected spatula in 0.05% Tween-80 (Sigma-P1754) solution [10,41]. The calculation of spores was done by using a hemocytometer [42,43]. Stock solutions were kept at 4 °C and used in serial dilution for making the desired concentration of entomopathogenic fungi.

### 2.2. Screening of Entomopathogenic Fungi

Three different entomopathogenic fungi were screened out against *P. xylostella*. Five concentrations (4 × 10⁸, 4 × 10^7^, 4 × 10^6^, 4 × 10^5^, 4 × 10^4^ spores/mL) were prepared (hit and trial method) while aqueous 0.05% Tween-80 (Sigma-P1754) was taken as control [41]. The application of entomopathogenic fungi was done by dipping the larvae in desired concentrations. After dipping, larvae were placed on filter paper for drying and then placed in disinfected plastic dishes (5 cm diameter) [34]. Fifteen larvae (3rd instar neonates) were exposed to each concentration. The experiment was repeated four times. A sufficient amount of diet was provided throughout the experimentation. Larval mortality was recorded every 24 h for seven days. Larvae without movements were considered dead.

### 2.3. Isolate Selection

Lethal and sublethal doses were calculated from the pre experimentation data. Isolate having the least LC_50_ with maximum mortality was selected for the downstream application.

### 2.4. Experimental Validation of Lethal (LC_50_) and Sublethal (LC_20_) Concentrations

Calculated lethal and sub-lethal concentrations were validated. Experimentation was carried out by following similar methodology described above. Each treatment included 15 larvae (3rd instar neonates) of *P. xylostella*. The experiment was replicated four times.

### 2.5. Entomopathogenic Fungi Effect on Hemocyte Concentration of P. xylostella 

The hemocyte concentration in *P. xylostella* larvae was calculated on lethal (LC_50_) concentration of *M. anisopliae* at different time points (24, 48, 72, and 96 h). Larvae were surface sterilized with ethanol (70%) and rinsed with double distilled water. Hemolymph was collected by dissection through proleg (30 larvae) using a sterilized blade and collected via glass capillary. Hemolymph was mixed with an equal amount of anticoagulant (98 mM NaOH, 186 mM NaCl, 17 mM Na2 EDTA, and 41 mM citric acid, pH 4.5). Hemocyte concentrations were quantified using a hemocytometer with 10 μL under a microscope [44]. The experiment was replicated four times.

### 2.6. PO and SOD Activity in P. xylostella Larvae

Hemolymph was collected from 30 treated larvae. The collection was done after 24, 48, 72, and 96 h post-infection. Collected hemolymph was diluted ten times and studied under a microplate reader (BIO-RAD). PO activity was checked using L-dihydroxyphenylalanine (L-DOPA) as the substrate on the initial linear increase in absorbance at 490 nm [23]. SOD activities were observed using respective kits following the manufacturer’s instructions (Suzhou Comin Biotech Co., Ltd., Suzhou, China). SOD activity was checked at a wavelength of 560 nm via a light reduction of nitro blue tetrazolium (NBT). NBT reduction (50%) is the quantity of enzyme for each unit of SOD. Units/mg protein was used for both enzyme activities.

### 2.7. Effect of M. anisopliae on the Expression of Immune-Related Genes in P. xylostella

After infection of *M. anisopliae*, quantitative real-time PCR (qRT-PCR) was used to investigate the expression of immune-related genes (Cecropin, Defensin, Attacin, and Spaetzle). Total RNA was extracted from hemolymph and reverse-transcribed in a 25-uL reaction according to the manufacturer guideline (TaKaRa, Beijing, China). After reverse transcription, qRT-PCR was done by using (Bio-Rad iQ2 optical system (BioRad) with SsoFast Eva Green Supermix (Bio-Rad, Hercules, CA, USA). The working program was set as 95 °C for 2 min, and 40 cycles of 95 °C for 5 s, and 60 °C for 10 s, melting curve from 65 to 95 °C [45]. The expression of *β-actin* was selected to normalize the expression of the immune-related genes according to the 2^−ΔΔCt^ method by Pfaffl, 2001 [46]. Three replicates were used in all experiments. Gene-specific primers were designed using Primer Premier 5. The gene sequences were subjected to Primer BLAST (www.ncbi.com) to check them for specificity The primers used are given in Table S1 [47].

### 2.8. Effects of Lethal Concentration of M. anisopliae on Biological Parameters of P. xylostella

The effects of lethal concentration of *M. anisopliae* was evaluated against *P. xylostella*. Each replication consists of 30 larvae of 3rd instar. For the treatment of fungi, the dip method was used. Petri dishes (diameter 5 cm) cleaned (70% ethanol) and air-dried for treating the larvae. Newly emerged cabbage leaves which were gently washed with double distilled water and air-dried served as larval diet. On emergence, the adults were paired (1 pair/cage) in plastic cages (cleaned with 70% ethanol and air-dried) for egg-laying. Sugar solution (10%) was provided as an adult diet. Whatman filter tape was used as an egg-laying pad. Eggs were counted under a microscope and were placed in plastic boxes (15 cm × 10 cm × 5 cm) for hatching. Data were collected every 12 h until the end of the experiment. Immobile larvae were considered dead and placed in a humid chamber for conidial growth observation [48].

### 2.9. Statistical Analysis

Mortality data were analyzed by using Probit analysis [49]. Abbott formula was used for the correction of mortality [50,51]. Lethal and sublethal concentrations for all entomopathogenic fungi were calculated by using SPSS 17.0 (SPSS Inc., Chicago, IL, USA) and Polo Pc (Petaluma, CA, Canada) [52]. One-way ANOVA was used for mortality data, means were separated by Tukey’s HSD test with a 5% level of significance (*p* < 0.05) [53]. Demographic data were analyzed by using Statistics 8.01 [53].

The hemocytes concentration and enzymatic activities (SOD and PO) after the treatment of *M. anisopliae* were analyzed by t-test. The relative expression of the selected immune genes was also analyzed via a t-test with a significance level set as *p* < 0.05.

## 3. Results

### 3.1. Screening of Entomopathogenic Fungi

**Figure 1:**
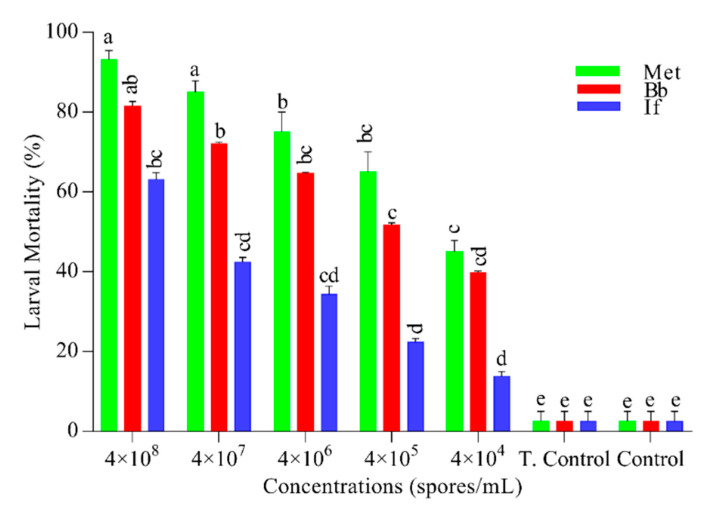
Larvicidal activity of entomopathogenic fungi. Mortality was recorded until seven days after every 24 h. Green, red, and blue bars represent the larval mortality of *Plutella xylostella*, after exposure to different concentrations of *Metarhizium anisopliae* (Met), *Beauveria bassiana* (Bb)*,* and *Isaria fumosorosea* (If) respectively. While T. Control is tween control (0.05% aqueous diluted) and in Control, distilled water was used. Error bars show 95% confidence intervals (CI). Letters indicate significant differences at *p* < 0.05.

**Figure 2:**
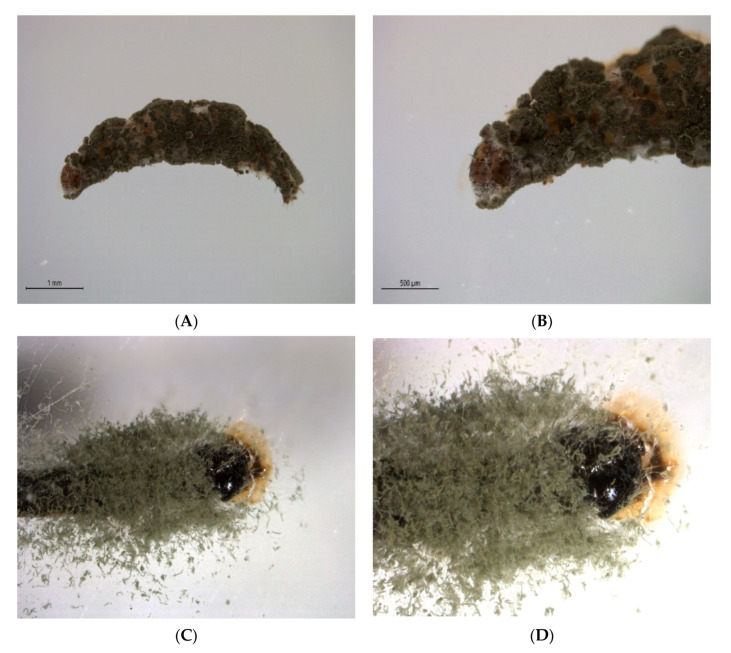
Conidial growth of *Metarhizium anisopliae* on larvae of *Plutella xylostella*. Larvae were placed in a humid chamber for confirmation of death due to fungi. (**A**) Conidial growth over the full body of the dead larvae. (**B**) Zoomed in the image of the conidia growing on the head of the dead larvae. (**C**) Conidial growth around the head and thorax of larvae. (**D**) Zoomed in the image of conidial growth adjacent to the head.

The results were highly concentration-dependent (Figure 1). *M. anisopliae* showed the highest mortality (93.13%) at 4 × 10⁸ (spores/mL) followed by *B. bassiana* (81.51%) while the least mortality (77.52%) was observed in *I. fumosorosea* (F = 89, DF = 4, *p* = 0.001). Mortality in control and tween control was insignificant. For confirmation of fungal pathogenicity, the carcasses were placed in a humid chamber for the growth of conidia (Figure 2).

### 3.2. Selection of Entomopathogenic Fungi

Based on pre experimentation data lethal (LC_50_) and sublethal (LC_20_) doses were calculated with (95% CL). *M. anisopliae* was selected on the basis of designed criteria (Table 2) as it showed the highest percent mortality and least LC_50_.

### 3.3. Experimental Validation of Lethal (LC_50_) and Sublethal (LC_20_) Concentrations of M. anisopliae

Lethal and sublethal concentrations of *M. anisopliae* were validated, showing 51.74% and 32.11% larval mortality respectively (Supplementary Figure S1).

### 3.4. Effects of M. anisopliae on Hemocyte Concentration of P. xylostella

**Figure 3:**
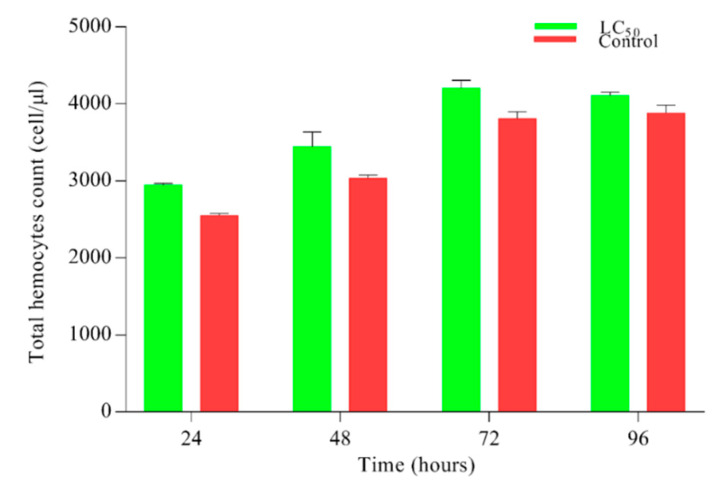
Hemocytes count in *Plutella xylostella* larvae after treatment with lethal (LC_50_) concentration of *Metarhizium anisopliae*. Green bars represent the hemocytes count of larvae under the pressure of LC_50_ while red bars show tween control (0.05% aqueous diluted) hemocytes count. Results were statistically non-significant *p* < 0. 071. Error bars show 95% confidence intervals (CI).

After infecting the larvae with lethal (LC_50_) concentration of *M. anisopliae*, results for hemocyte count are shown (Figure 3). Non-significant (0.071) results were observed regarding the hemocytes count on all time points.

### 3.5. Effects of M. anisopliae (LC_50_) on the Activity of PO and SOD in Larvae of P. xylostella

**Figure 4:**
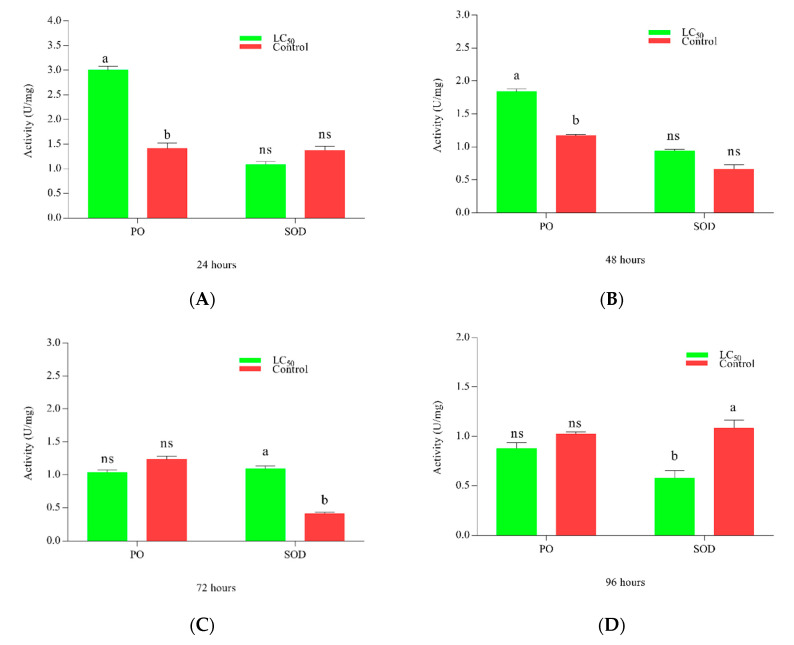
Activity of phenoloxidase (PO) and superoxide dismutase (SOD) activity in *Plutella xylostella* against LC_50_ of *Metarhizium anisopliae*. Time-dependent activity is shown in the figure, the green color bar shows the activity of enzymes whereas the red bar represents the control. (**A**–**D)** shows the activities of enzymes after 24, 48, 72, and 96 h respectively. Error bars show 95% confidence intervals (CI). Letters indicate significant differences at *p* < 0.005. (ns = non-significant).

The activities of immune enzymes PO and SOD against LC_50_ of *M. anisopliae* are shown in Figure 4. PO activity peaked at 24 h, equaling control at 48 h while decreasing later on 48 and 72 h. SOD activity amplified around 72 h post-treatment. Figure 4A–D shows the activity of enzymes at 24, 48, 72, and 96 h respectively.

### 3.6. Effect of Lethal Concentration of M. anisopliae on Immune Genes of P. xylostella

**Figure 5:**
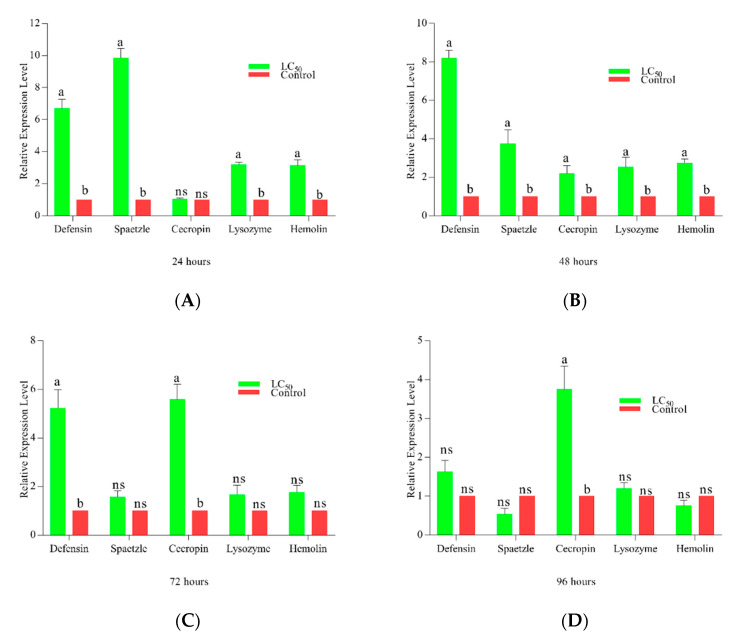
Immune genes expression in *Plutella xylostella* against LC_50_ of *Metarhizium anisopliae*. Time-dependent activity is shown in the figure, the green color bar shows the activity of gene at LC_50_ of *M. anisopliae* while the red bar shows the control. (**A**–**D**) shows the activities of enzymes after 24, 48, 72, and 96 h respectively. Error bars show 95% confidence intervals (CI). Letters indicate significant differences at *p* < 0.001. (ns = non-significant).

The expression of all immune genes was predominantly time-dependent (Figure 5), with a significance level of 0.001. Defensin showed a substantial increase in expression after 24 h of treatment compared to control followed by a decrease at 72 h ultimately approaching non-significant expression at 96 h. Spaetzle showed the highest expression at 24 h post-treatment among all the genes, gradually decreasing after 48 h and keep on decreasing until 96 h. Cecropin expression level increased significantly until 72 h while decreasing later at 96 h. Hemolin and lysozyme, both genes showed similar trends at 24 and 48, but both showed non-significant activity after 72- and 96-h post-treatment

### 3.7. The Lethal Concentration of M. anisopliae Affects the Biological Parameters of P. xylostella

**Figure 6:**
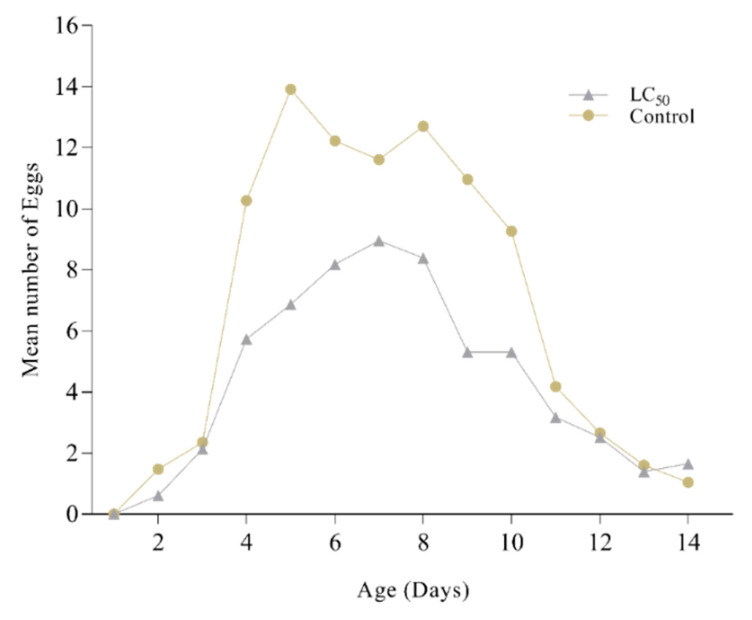
Mean number of eggs from *Plutella xylostella* after treatment with *Metarhizium anisopliae.* Grey dots show the mean numbers of eggs laid by *P. xylostella* after treatment by *M. anisopliae* while yellow dots show the control group.

*M. anisopliae* (LC_50_) disturbs the biological parameters of *P. xylostella* (Table 3). After the application of lethal concentration on 3rd instar, a significant reduction in larval duration (1.10 days) was observed compared to control (2.4 days) (*p* > 0.05). A similar trend was followed by 4th instar. A considerable decrease in percent pupation was also reported in the infected population with only 64.21% larvae arriving at the pupal stage compared to 93.51% in control. Prolonged pupal duration (5.22 days) was followed by reduced adult emergence (61.47%) with a ratio of 1:2 sex ratio (M: F). The pre-oviposition period (APOP) in adult females increased to 1.92 days compared to 1.01 days in control. Fecundity was also significantly affected in treated females where the mean number of eggs laid was reduced to 101.55 eggs/female in comparison to 192.55 eggs/female control. A noteworthy effect was also seen on egg hatching (first filial generation). The daily egg production rate is shown in Figure 6, where the number of eggs in control is significantly higher than the treated group.

## 4. Discussion

*P. xylostella* is a notorious pest of cruciferous crops [20]. IPM strategies employed to control this pest has been focused primarily on the use of chemical insecticides [8]. Compared to synthetic insecticides, pathogenic fungi are promising biocontrol agents for various insect pests and exhibit efficient capabilities for insecticide-resistant pests with fewer environmental hazards [2,16]. The study presented here evaluated three different types of entomopathogenic fungi for larval mortality in *P. xylostella*. Our results found that *M. anisopliae* could efficiently infect *P. xylostella,* and cause significant mortality compared to the rest, suggesting the potential of this entomopathogenic fungus for the pest control [3]. The difference in pathogenicity could be due to the fact that some fungal species germinate and penetrate more rapidly compared to others. Besides, an increase in the production of secondary metabolites to resist antifungal compounds is also the primary adaptive behavior of a potent entomopathogenic fungus [54,55,56]. Conversely, insect cuticle, a hydrophobic surface, acts as the first line of defense against invading fungi [33,57]. Entomopathogenic fungi encounter this barrier by producing hydrophobin proteins that synergies with enzymes, leading to infection and causing the death of the insect [33,57]. The mortality caused by *M. anisopliae* was highly concentration-dependent as previously described in other insect pests [10,43,58]. Insects possess an innate immune system, a dynamic and instantaneous mechanism against pathogenic infections [59]. The suppression of immune responses is one of the major mechanisms which govern the outcome of an interaction between pathogen and host [60,61].

Hemocytes, upon invasion, play a vital role to defuse the pathogen activities by employing different biological processes such as phagocytosis and encapsulation [62,63]. Arthropods can produce various types of hemocytes depending upon the type of infection they face [64]. Studies have revealed that the number and types of hemocytes varied when infected with different strains and strengths of *Metarhizium* spp. [65].The ability of a pathogenic fungi to overcome host hemolymph also represents its virulence to the host [66]. The study presented here showed a change in hemocyte numbers under pressure from the lethal concentration of *M. anisopliae*, which could be due to evasion and overcoming hemolymph defense systems by entomopathogenic fungus. However, this change was not statistically significant, supported by similar findings in previous studies [67].

The enzymatic responses are a key constituent of the insect immune system under various stressful environments, reflecting physiological changes in the host. Enzymes such as PO play a crucial role in wound deposition, encapsulation of pathogens, and most importantly activation of immune pathways [68]. The increased PO activity strengthens the ability of the immune system to counter xenobiotics [69,70], while its inhibition suggests pathogenic fungi may overcome the immunity of PO. The current study depicted a time-based trend in PO activity rising to a maximum between 24 and 48 h while gradually decreasing at 72 and 96 h, indicating that *M. anisopliae* steadily get the better of the host defense system [69]. Similarly, SOD an antioxidant defense enzyme, also reported varying trends demonstrating that the physiological activities of *P. xylostella* larvae were distressed following the infection by fungi [71,72]. Insect immune responses are governed by various immune genes expressed at different time points. Scientists have been able to identify 1000 immune-related genes in *P. xylostella* when targeted by entomopathogenic fungi [34]. Likewise, our study found out key genes (spaetzle, defensin, cecropin) involved in immune responses against entomopathogenic fungi were exceedingly time-dependent, showing varying expression levels.

*M. anisopliae* produces secondary metabolite destruxin, a compound capable of evading insect cellular and humoral immune responses, troubling the demographic parameters of the pest before its eventual death [73,74,75,76]. Disturbances in biological parameters of various insect pests have been reported when infected with entomopathogenic fungi [10,11,26,61,62], which strengthens our findings where a distress in biological cycles are reported. During the invasion of pathogens (*M. anisopliae*) the body temperature of insects rises to encounter the infection but this, in turn, affects the larval stadium [77] supporting the current findings where reduced larval duration was observed. *M. anisopliae* absorbs hemolymph sugar content from tracheoles, reducing nutrients and leading toward early pupation, prolonged pupal duration [35,78], reduced percent pupation, and percent emergence in different insect pests [2,79,80] supporting the findings reported in a study presented here. Besides intrusion in immature stages, the adults are also affected in the form of shorter life span, an observation supported by previous findings where reduced adult age and fecundity were reported [43,57,80,81].

## 5. Conclusions

The study conducted demonstrates the interaction of fungal pathogen *M. anisopliae* with immune responses of *P. xylostella* and potentially overcoming it by causing the disturbance in its demography eventually killing the host. Hence, *M. anisopliae* can become a potent prospect for the control of this pest. The study will provide a basic hence important information for further field and semi field experimentation.

## Figures and Tables

**Table 1:** Isolates of entomopathogenic fungi from Pakistan.

Fungi	Source	Location (Pakistan)	Coordinates
*Metarhizium anisopliae*	Soil	Multan, Punjab	30°05′11.65″ N 71°39′15.65″ E
*Beauveria bassiana*	Soil	Multan, Punjab	30°05′11.65″ N 71°39′15.65″ E
*Isaria fumosorosea*	Soil	Multan, Punjab,	30°05′11.65″ N 71°39′15.65″ E

**Table 2:** Lethal and sublethal concentrations of fungi against *Plutella xylostella.*

Fungi	LC_50_	LC_20_	Slop ± SE	χ^2^	*p*-Value	df
*Metarhizium anisopliae*	6.2 × 10^4^	2.3 × 10^2^	0.29 ± 0.044	2.1	0.001	4
*Beauveria bassiana*	9.3 × 10^5^	3.1 × 10^3^	0.38 ± 0.044	1.1	0.003	4
*Isaria fumosorosea*	7.9 × 10^6^	4.6 × 10^4^	0.42 ± 0.046	1.8	0.002	4

**Table 3:** Biological parameters of *Plutella xylostella* after treatment of *Metarhizium anisopliae.*

Parameters	*M. anisopliae* (LC_50_)	Control
	Means ± SE	Means ± SE
Mortality (%)	51.34 ± 1.25 ^a^	5.4 ± 0.10 ^b^
L3 (days)	1.10 ± 0.21 ^b^	2.14 ± 0.17 ^a^
L4 (days)	1.72 ± 0.11 ^b^	2.59 ± 0.12 ^a^
Percent pupation	64.21 ± 2.46 ^b^	93.51 ± 3.11 ^a^
Pupal duration (days)	5.22 ± 0.71 ^a^	3.80 ± 0.27 ^b^
Adult emergence (%)	61.47 ± 3.41 ^b^	92.11 ± 3.57 ^a^
Female longevity (days)	6.87 ± 0.98 ^b^	10.11 ± 1.98 ^a^
Male longevity (days)	8.11 ± 1.27 ^b^	12.38 ± 2.8 ^a^
Sex ratio (M:F)	1:2 ^ns^	1:2 ^ns^
APOP	1.92 ± 0.04 ^ns^	1.01 ± 0.07 ^ns^
Fecundity (eggs/female)	101.55 ± 2.54 ^b^	192.55 ± 3.21 ^a^
Egg hatching (%)	60.2 ± 3.44 ^b^	94.22 ± 2.98 ^a^

L3 = 3rd instar larval duration; L4 = 4th instar larval duration; APOP = adult pre-oviposition period of female adult; means in the same row followed by the same letter are not significantly different (*p* > 0.05).

## Supplementary Materials

The following are available online at https://www.mdpi.com/2075-4450/11/10/694/s1, Figure S1: Experimental validation of lethal (LC50) and sublethal (LC20) concentrations of Metarhizium anisopliae. Error bars show 95% confidence intervals (CI), Table S1: Primers used in this study.
