# Supplementary material for: Metarhizium Anisopliae Challenges Immunity and Demography of Plutella xylostella
Source: Insects. 2020 Oct 13;11(10):694. doi: 10.3390/insects11100694 (PMC7600445; doi:10.3390/insects11100694)
Supplement: Supplementary file 1 [file insects-11-00694-s001.pdf]

Supplementary Material

Supplementary Table 1. Primers used in this study

Name		Primer sequences
β-actin	*F*	GGAACGATACAGAGAAGATTA
	R	TGGAACGCTTCACGAATTTGCG
Cecropin	*F*	*GTCGTGGCCATATGCTGTGT*
	R	*GTCGTCGGACAAGCATCCAG*
Lysozyme	*F*	*GCGAGTTGATAACTGACGACAT*
	R	*GGCACTTGTTCTTCCATCCATA*
Hemolin	*F*	*AATAGAAGCTACCACAACGCCAGAG*
	R	*TAACGCAGATGCCGTAAGTGTAACC*
Spaetzle	*F*	*CCAACAGCATCATACCGAGC*
	R	*TCCTGACATCGGCTTCCAAT*
Defensin	*F*	*ACAGGAGACAGTGGTTGAGGAGTC*
	R	*TTGTATCTTCAGTGGCGTCTTCGTAC*

F′= Forward, *R′* Reverse

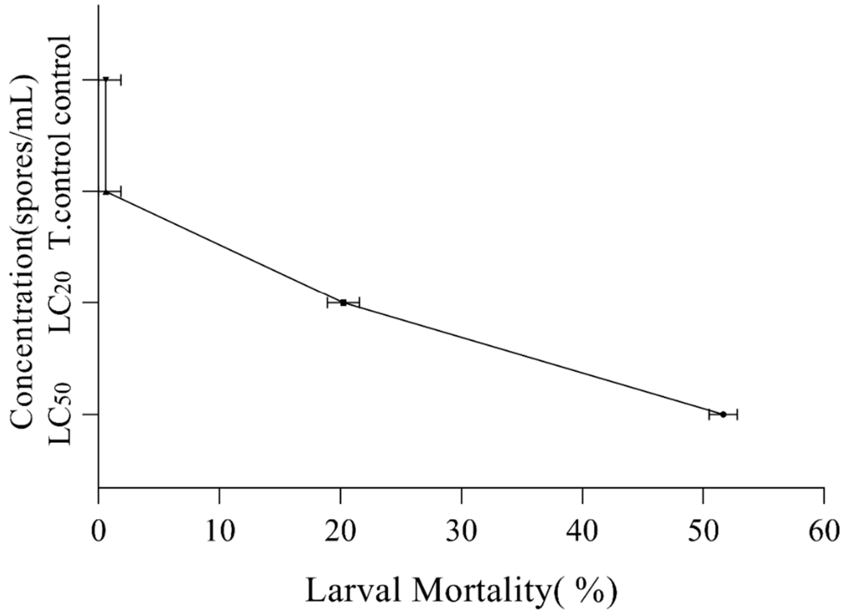

Supplementary Figure 1: Experimental validation of lethal (LC_{50}) and sublethal (LC_{20}) concentrations of *Metarhizium anisopliae*. Error bars show 95% confidence intervals (CI).
